# Pilot study of a model-based approach to blood glucose control in very-low-birthweight neonates

**DOI:** 10.1186/1471-2431-12-117

**Published:** 2012-08-07

**Authors:** Aaron J Le Compte, Adrienne M Lynn, Jessica Lin, Christopher G Pretty, Geoffrey M Shaw, J Geoffrey Chase

**Affiliations:** 1 Department of Mechanical Engineering, University of Canterbury, Christchurch, New Zealand; 2 MBChB, FRACP, Neonatal Department, Christchurch Women’s Hospital, Christchurch, New Zealand; 3 Department of Medicine, University of Otago, Otago, New Zealand; 4 MbChB, FJFICM, Department of Intensive Care, Christchurch Hospital, Christchurch School of Medicine and Health Science, University of Otago, Otago, New Zealand

**Keywords:** Hyperglycemia, Premature birth, Insulin, Control, Insulin sensitivity

## Abstract

**Background:**

Hyperglycemia often occurs in premature, very low birthweight infants (VLBW) due to immaturity of endogenous regulatory systems and the stress of their condition. Hyperglycemia in neonates has been linked to increased morbidities and mortality and occurs at increasing rates with decreasing birthweight. In this cohort, the emerging use of insulin to manage hyperglycemia has carried a significant risk of hypoglycemia. The efficacy of blood glucose control using a computer metabolic system model to determine insulin infusion rates was assessed in very-low-birth-weight infants.

**Methods:**

Initial short-term 24-hour trials were performed on 8 VLBW infants with hyperglycemia followed by long-term trials of several days performed on 22 infants. Median birthweight was 745 g and 760 g for short-term and long-term trial infants, and median gestational age at birth was 25.6 and 25.4 weeks respectively. Blood glucose control is compared to 21 retrospective patients from the same unit who received insulin infusions determined by sliding scales and clinician intuition. This study was approved by the Upper South A Regional Ethics Committee, New Zealand (ClinicalTrials.gov registration NCT01419873).

**Results:**

Reduction in hyperglycemia towards the target glucose band was achieved safely in all cases during the short-term trials with no hypoglycemic episodes. Lower median blood glucose concentration was achieved during clinical implementation at 6.6 mmol/L (IQR: 5.5 – 8.2 mmol/L, 1,003 measurements), compared to 8.0 mmol/L achieved in similar infants previously (p < 0.01). No significant difference in incidence of hypoglycemia during long-term trials was observed (0.25% vs 0.25%, p = 0.51). Percentage of blood glucose within the 4.0 – 8.0 mmol/L range was increased by 41% compared to the retrospective cohort (68.4% vs 48.4%, p < 0.01).

**Conclusions:**

A computer model that accurately captures the dynamics of neonatal metabolism can provide safe and effective blood glucose control without increasing hypoglycemia.

**Trial Registration:**

ClinicalTrials.gov registration NCT01419873

## Background

Hyperglycemiaoccurs in many premature neonates. The threshold for hyperglycemia differs between studies, but prevalence of hyperglycemia has been reported in 57% of extremely low birthweight (ELBW) infants [1] and 32–86% of very low birthweight (VLBW) infants [2,3].An increasing body of literature links hyperglycemia to worsened outcomes in premature neonates, but there have been no studies of sufficient power to demonstrate whether hyperglycemia itself is harmful, or is merely a reflection of disease severity.

Loss of glucose regulation can be caused by clinical stress, leading to a rise in hepatic gluconeogenesis as well as a reduction in insulin sensitivity [4]. At the same time, premature VLBW infants have reduced ability to produce insulin [2]; defective beta-cell processing of pro-insulin (which is 10–16 times less active than insulin) to insulin [5]; an inability to suppress hepatic glucose production in response to glucose infusion [6]; and, finally, a decreased uptake of glucose secondary to a limited mass of insulin-sensitivity tissues such as muscle and adipose tissue [7].

Neonatal units will differ in their approach to the management of hyperglycemia. There is no strict definition for hyperglycemia, but, it is generally regarded as a blood glucose (BG) exceeding 10 mmol/L [8]. There is no consensus on the threshold for intervention, which reflects the lack of reliable evidence upon which to base management decisions [8]. Glucose restriction can be used to control high blood glucose levels [9]. However, this approach also deprives the neonate of crucial energy required to promote growth [2]. A small number of prospective trials have used insulin infusions to treat hyperglycemia and/or promote growth [10-18]. Positive outcomes of insulin infusion have included reduced proteolysis, improved glucose tolerance, increased IGF-I levels and improved caloric intake and weight gain. The American Academy of Paediatrics has supported the use of insulin since 1985[19]. However, larger trials of insulin usage in both neonates and adults have been confounded by increased rates of hypoglycemia. Recently, the NIRTURE trial used a fixed dose of insulin on day 1and modulated glucose infusions versus standard care and found an increased rate of hypoglycemia in the treatment group. This studywas stopped early [10]. There have also only been two other small randomized controlled trials with a total of 47 patients that have compared different managementoptions forhyperglycemia in this group of patients [12,20].

All reported neonatal insulin infusion trials have used either protocols that fixed insulin dosing to weight or other factors [21], or clinician judgment to determine insulin infusion rates. It is well reported that preterm infants can show great variety in terms of sensitivity to exogenous insulin infusions [2]. This suggests that the use of insulin will increase the risk of hypoglycemia, unless variability in sensitivity to insulin is explicitly taken into account. Thus, an insulin dosing strategy to achieve both goals of reduced blood glucose levels and safety from hypoglycemiashould a) estimate a patient’s particular level of response to insulin, b) account for any potential changes in sensitivity to insulin over time, and c) adapt dosing accordingly to cater for individual patients.

Model-based systems attempt to control BG by using a mathematical representation of the glucose-insulin system to quantify a patient’s insulin sensitivity and track changes over time [22,23]. Databases of sensitivity to insulin can be created using retrospective data from babies that have received insulin [22]. This information can be interrogated using stochastic tools to observe how the level of response to insulin in babies varies both between patients and over time [24]. Forecasts of potential changes in sensitivity to insulin can be generated for a patient at a particular point in time to compute the likely impact on BG levels for a given prospective dose of insulin [24]. Thus, the dose of insulin can be optimized to balance the requirements of lowering blood glucose levels whilst reducing the risk of hypoglycemia [25,26].

Blood glucose control using model-based methods has been applied successfully in limited adult clinical trials [27] and large-scale clinical implementation [28], which reduced both blood glucose levels and hypoglycemia using a combined insulin and nutrition protocol [29]. The success of this model-basedsystem in adults suggests that such an approach could also provide a useful tool for metabolic management in neonates. This study presents the first trial of model-based glycemic control in this unique neonatal patient population.

## Methods

### Study population

This study was approved by the Upper South A Regional Ethics Committee, New Zealand (ClinicalTrials.gov registration NCT01419873). Infants who met eligibility criteria were recruited between August 2008 and June 2011 from the neonatal intensive care unit (NICU) at Christchurch Women’s Hospital. Inclusion criteria were birthweight < 1,500 g, blood glucose concentration ≥ 10 mmol/L and a clinical decision to commence an insulin infusion. Infants who were not expected to survive were excluded. Written parental consent was obtained for each study participant during the pilot study phase. Data from a retrospective cohort of 21 infants that received insulin in 2005–2006 in the same NICU, before the introduction of the computer-based system, were collected for comparison.

### Clinical protocols

This study was conducted in two parts: a series of short-term trials that intensively monitored infants to assess safety and effectiveness; and long-term trials that implemented the system into daily clinical practice. The study period during short-term trials was up to 24 hours, and most patients enrolled were already receiving insulin infusions. Blood glucose concentrations were measured every 1 to 3 hours (maximum 12 measurements per day). Long-term trials covered the entire period of insulin usage for an infant and blood glucose concentrations were generally measured at 2–4 hour intervals at the attending clinician’s discretion. The same computer system was used for both parts of the study.

The insulin infusion rate was adjusted as determined by the model-based controller after each BG measurement. Occasionally, blood gas measurements were taken for other clinical reasons and provided additional blood glucose concentration data. This extra data was also used to update the insulin infusion rate. Blood was drawn from an in-situ arterial line if present, otherwise from capillary samples and analyzed with a Bayer 850 blood gas analyzer (Bayer AG, Leverkusen, Germany).

**Figure 1:**
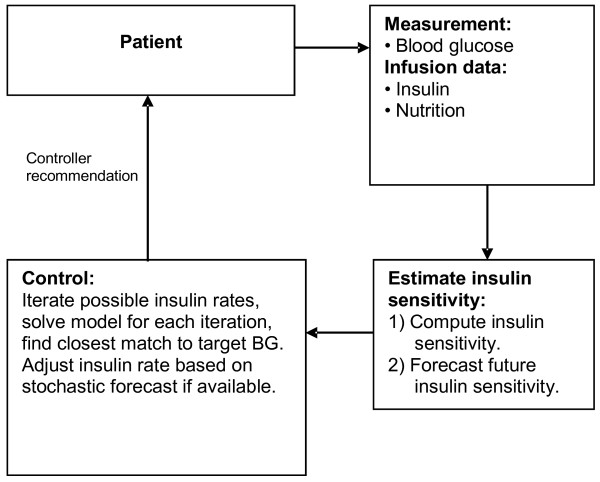
Controller implementation overview.

The blood glucose concentration profile, together with the insulin and nutrition data, was used by the computer algorithm to determine insulin infusion rates to reach the target range of 4 to 7 mmol/L (Figure 1). Model “insulin sensitivity” was estimated from the clinical data in real-time to identify the current metabolic state of the infant [22]. The controller used the fitted insulin sensitivity value to iterate through several possible insulin infusion rates and forecast a blood glucose concentration 1 to 4 hours ahead, depending on the time of the next planned measurement. The insulin infusion rate that was predicted to achieve a blood glucose concentration closest to the target was selected.

A stochastic insulin sensitivity model [24] was used to provide confidence limits around the forecasted blood glucose concentration, and the selected insulin infusion rate was adjusted to ensure that the lower 5% probability limit of the forecasted blood glucose concentration was > 4 mmol/L. Further details on the model identification, control methodology and stochastic modeling of insulin sensitivity are published elsewhere [24,26,30].

The computer system uses entered information on insulin rates, dextrose input rates from IV and enteral sources and prior blood glucose concentrations to determine the insulin sensitivity level of the patient [30]. This insulin sensitivity parameter represents the level of glycemic response the infant has been exhibiting to exogenous insulin over the last few hours. Thus, lower observed insulin sensitivity would result in recommendations of relatively higher insulin rates and vice-versa. A stochastic model is used to determine the potential changes in sensitivity to insulin in the upcoming hours based on observed changes in sensitivity to insulin in retrospective data [24]. The current BG, rates of dextrose inputs, level of sensitivity to insulin and forecasted changes in sensitivity to insulin are used by the computer model to select an insulin rate that balances the goals of achieving BG within the target range whilst limiting the potential for hypoglycemia [25,31]. Incorporating information about nutrition inputs allows insulin dosages to be scaled accordingly to provide control for infants receiving higher and lower amounts of calories.

Infants received most nutrition via parenteral solutions containing 10–12.5% dextrose. Several infants also received expressed breast milk (EBM), and some infants received morphine and dobutamine infusions prepared using 5% dextrose. All sources of glucose and any hour-to-hour changes were considered by the model-based controller algorithm when recommending insulin infusion rates.

Insulin was given via intravenous lines using Alaris CC pumps (Alaris, San Diego, California, USA) as a continuous infusion. The concentration of insulin was [5 x weight (kg)] U made up to 20 mL with 0.9% saline solution to achieve a concentration of 0.25 U/kg/mL. Insulin tubing was flushed with this solution to minimize subsequent adsorption of insulin to the tubing [32]. New insulin infusion rates were determined after every BG measurement and a neonatal clinician chartedevery change in insulin infusion rate before adjusting the pump, which is standard practice. The maximum allowable insulin infusion rate was restricted to 0.5 U/kg/hr for safety.

The retrospective group received blood glucose control using insulin infusions using the sliding scale presented in Table 1 as a guideline. Actual insulin rates used in these patients were left to the discretion of the attending clinician, who could deviate from the suggested scale if desired. Thus, there was no explicit BG target, but the clinical practice of this unit was to aim to maintain BG within the 5 – 8 mmol/L range, and results from these patients are reported to provide context to represent typical clinical practice in thus unit prior to the introduction of model-based control.

**Table 1:** Retrospective control insulin sliding scale.Insulin started at 0.2 ml/hr = (0.05units/kg/hr using standard insulin dilution)

**BG**	**Insulin adjustment**
> 20 mmol/L	0.4 ml/hr (0.1 U/kg/hr)
15 – 20 mmol/L	0.3 ml/hr (0.075 U/k/hr)
10 – 15 mmol/L	0.2 ml/hr (0.05 U/kg/hr)
< 5 mmol/L	STOP

Manipulations were recommended based on BG concentration.

## Results

Eight infants were included in the short-term trial with median birth gestation of 25.6 weeks and median birthweight of 745grams. Infants were enrolled at 1 to 9 days of age (Table 2). Insulin was already being used on 6 of the 8 short-term trial patients before the 24-hour trial period, and the median time of insulin usage before these trials was 7.4 hours. The 27 long-term trials were performed on 22 patients with median birth gestation of 25.4 weeks and median birthweight of 760 grams. The retrospective cohort comprised 21 patients with median gestation of 26.6 weeks and median birthweight of 845 grams. Babies with birthweight between 1,000 – 1,500 grams formed a minority of the patients in these cohorts, with 4 babies in the long-term study, 2 babies in the retrospective cohort and none of the babies in the short-term study in this weight range.

**Table 2:** Clinical details of study populations

	**Short-term (N = 8)**	**Long-term (N = 22)**	**Retrospective (N = 21)**
Gestational age at birth (weeks)	25.6 [24.9 – 26.4]	25.4 [25.0 – 26.8]	26.6 [25.4 – 27.7]
Weight at birth (grams)	745 [681 – 814]	760 [601 – 925]	845 [800 – 904]
Age at start of trial (days)	6.6 [3.6 – 7.7]	3.6 [1.5 – 6.4]	n/a

Data presented as median [inter-quartile range].

Clinical blood glucose results during the study are presented in Table 3. Linear interpolation between BG measurements was used to provide hourly estimates of BG concentrations. Thus, percentages of measurements within reported ranges represent an estimate of the time spent within the specified range to ensure an equal and fair comparison across datasets with different measurement frequencies. The control system was used for a total of 226 hours in short-term trials and 3,168 hours in long-term trials, and 3,571 hours of control were available for the retrospective cohort.A mixture of arterial and capillary BG samples were used in the long-term and retrospective data sets as presented in Table 3. Four long-term patients and two retrospective patients had multiple episodes of insulin usage during their neonatal intensive care stay. The whole-cohort median BG during long-term trials was 6.6 mmol/L,which was within the target 4 – 7 mmol/L range. By comparison, the retrospective median BG level was a more conservative 8.0 mmol/L(p < 0.01, Mann–Whitney test). The short-term trials spent a larger proportion of time reducing blood glucose from hyperglycemia levels, so the higher median of 7.4 mmol/L was expected, and blood glucose results are not directly comparable between short-term trials and either the long-term or retrospective results.

**Table 3:** Clinical blood glucose control variables during trials and comparison with retrospective control

**Whole cohort statistics**	**Short-term**	**Long-term**	**Retrospective**
Number of episodes	8	22	21
Total hours	226 hours	3168 hours	3571 hours
Number of BG measurements	117	1003	1091
BG sampling site: arterial/capillary	100%/0%	27%/73%	45%/55%
BG median [IQR] (mmol/L)	7.4 [6.2 - 9.4]	6.6 [5.5 - 8.2]	8.0 [6.6 - 9.4]
% BG within 4.0 - 7.0 mmol/L	40.9	53.7	29.5
% BG within 4.0 - 8.0 mmol/L	63.9	68.4	48.4
% BG > 10 mmol/L	22.6	11.7	19.2
% BG < 4.0 mmol/L	0.9	4.0	2.7
% BG < 3.0 mmol/L	0.0	0.5	0.4
% BG < 2.7 mmol/L	0.00	0.25	0.25
Median insulin rate [IQR] (U/kg/hr)	0.058 [0.038 - 0.107]	0.033 [0.028 - 0.040]	0.025 [0.010 - 0.045]
Median glucose rate [IQR] (mg/kg/min)	8.2 [7.1 - 9.4]	7.9 [6.1 – 9.3]	8.4 [6.3 - 9.1]
**Per-patient statistics (presented as median [IQR])**			
Number of BG measurements	15.0 [13.0 - 17.5]	27.0 [15.0 - 44.8]	36.0 [21.0 - 54.0]
Initial BG (mmol/L)	11.4 [7.5 - 12.3]	11.4 [9.4 – 13.4]	8.9 [5.7 – 9.9]
Time between measurements (hours)	2.0 [2.0 - 2.1]	3.0 [2.8 - 3.3]	3.2 [2.6 - 4.0]
%BG within 4.0-7.0 mmol/L	41.4 [14.4 - 60.8]	60.4 [38.2 – 72.7]	29.6 [20.4 - 38.5]
%BG within 4.0-8.0 mmol/L	58.8 [48.3 - 75.9]	74.1 [57.7 - 84.2]	47.7 [42.9 - 55.7]
%BG < 4.0 mmol/L [IQR]	0.0 [0.0 - 0.0]	4.8 [3.1 - 9.3]	2.8 [0.0 - 5.4]
%BG < 3.0 mmol/L [IQR]	0.0 [0.0 - 0.0]	0.0 [0.0 - 2.0]	0.0 [0.0 - 0.2]
%BG < 2.7 mmol/L [IQR]	0.0 [0.0 - 0.0]	0.0 [0.0 - 0.7]	0.0 [0.0 - 0.0]
Dextrose rate (mg/kg/min)	1.2 [0.3 - 1.6]	5.2 [0.6 - 14.0]	4.8 [1.9 - 6.7]
EBM (mL/kg/day)	4.6 [1.0 - 5.7]	15.8 [2.3 - 41.9]	16.7 [5.4 – 27.2]
Patients that received EBM	6 (75%)	19 (86%)	18 (86%)
Proportion of dextrose via EBM (%)	1.2 [0.3 - 1.7]	11.0 [1.5 - 21.4]	5.5 [1.9 - 7.2]
Insulin rate (U/kg/hr)	0.058 [0.046 - 0.088]	0.033 [0.028 - 0.040]	0.025 [0.010 - 0.045]
Insulin sensitivity x 10^-3^ (L/[mU.min])	1.28 [0.48 – 1.86]	1.73 [1.25 - 2.65]	1.93 [1.40 – 2.58]

Blood glucose (BG) results are resampled hourly.

Safety from hypoglycemia (BG < 2.7 mmol/L) was present despite the lower achieved blood glucose levels during the trials. No hypoglycemic events were recorded during the short-term trials, and there was no significant difference in the low incidence of hypoglycemia between the long-term trials and retrospective control dataat a rate of 0.25% of resampled measurements each (p = 0.51, Fisher’s Exact test).

The percentage of BG within the computer control target 4.0 – 7.0 mmol/L range was 82% higher for the long-term cohort compared to retrospective control (53.7% vs. 29.5%, p <0.01, Chi-squared test). The wider 4.0 – 8.0 mmol/L band covers covers both computer-control and retrospective target ranges, and was 41% higher for computer control(68.4% vs. 48.4%, p < 0.01, Chi-squared test). Increased time within target ranges was consistent across patients, where the per-patient medians of BG within the 4.0-7.0 mmol/L and 4.0-8.0 mmol/L bands were consistently higher for model-based control (60.4% vs. 29.6% for 4.0 – 7.0 mmol/L band, p <0.01, Mann–Whitney test and 74.1% vs. 47.7% for 4.0 – 8.0 mmol/L band, p <0.01, Mann–Whitney test).

Infants during the trials showed large variations in response to insulin. The 90% range of per-patient median insulin sensitivity showed a 6.6x spread during the short-term trials and greater than 10x spread during the long-term trials. Thus, a particular infant may exhibit an over 10x stronger response to insulin than another infant. Additionally, the hour-to-hour changes may be even larger than the comparison of median sensitivity levels. Clinical dextrose and EBM usage also showed significant variation between patients, reflecting individual clinical condition and thus a wide range of insulin infusion rates were used by the control system.

**Figure 2:**
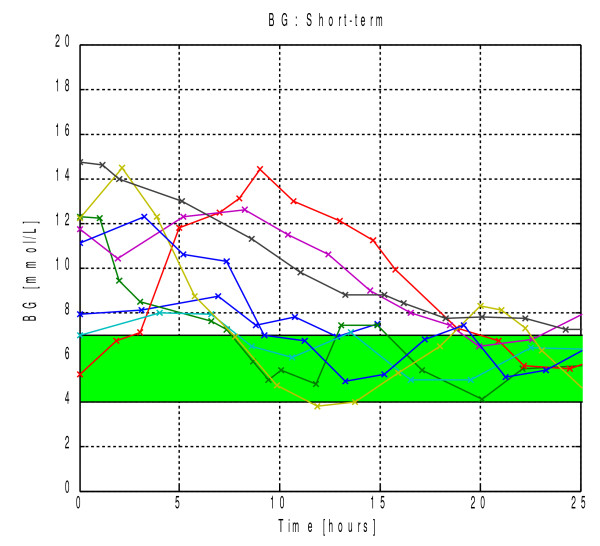
**BG concentration and estimated insulin sensitivity during short-term, long-term computerized insulin dosing trials and retrospective control.** For the short-term trials each line in the top row of plots represents blood glucose concentration for each patient. The shaded region represents the 4–7 mmol/L target band. Each line in the bottom row of plots represents the evolution of sensitivity to exogenous insulin for each patient. For the long-term and retrospective patients summary boxplots are presented for each day of control.

Figure 2 graphically presents the blood glucose concentrations and quantified insulin sensitivity during the trials. The short-term trials showed a uniform response of blood glucose approaching the target band over the approximately 24-hour trials. Over this time period insulin sensitivity was generally relatively constant, yet each patient had a unique value. The long-term blood glucose results showed a general tightening and approach to the target band with less incidence of blood glucose greater than 10 mmol/L compared to retrospective data. The long-term insulin sensitivity results showed significantly greater variability observed over the longer time scale. Additionally, any periods of BG below the target band during the long-term clinical implementation trials were generally brief and resolved quickly.

The model-based insulin dosing application was entirely run by clinical staff during the long-term study. The median time between BG measurements per patient during the long-term study was similar to retrospective patients on insulin at 3.0 versus 3.2 hours respectively. The system has been accepted for on-going use in this neonatal unit.

## Discussion

This study compared computer-based control of BG using insulin infusions against clinical control before the introduction of the system. The retrospective clinical control group had a generally higher target than the computer-controlled group. It is completely feasible that even though model-based control targeted a lower BG range it may have had no difference on BG concentrations compared to the previous methods of BG control. The results presented indicate that the model-based system achieved lower BG levels and greater time of BG spent in the desired target range, without excessive hypoglycemia.

Hyperglycemia has been linked to worsening outcomes for premature infants [1,33-36], but there is currently no best-practice approach to its management. Great inter-patient heterogeneity is a hallmark of neonatal glucose metabolism making safe, adequate control difficult [2,13]. Even within this relatively small study population a 10-fold spread of insulin sensitivity was computed during long-term trials. Additionally, the inter-patient variation in sensitivity to insulin observed in the short-term trials, presented in Figure 2, highlight this wide range of response between individuals. Thus, fixed insulin protocols based on weight or other patient parameters are not likely to accurately account for this level of inter-patient variability, and an adaptive protocol, as presented here, may provide better, safer control.

Model-based control provides real-time identification of insulin sensitivity, and its evolution with time. However, identification of insulin sensitivity relies on the availability of blood glucose concentration measurements. One to four hourly measurements were used in this study, based on simulation results [26], as a compromise between accurate metabolic identification and nursing/patient burden, magnified in premature neonates with limited blood volumes. Frequent glucose sampling has been shown to be an important precursor for tight glycemic control [26]. Some insulin infusion studies in premature neonates used longer measurement and intervention intervals of up to 6 hours [10], which may have contributed to the difficulty of achieving glycemic control [37]. Continuous glucose monitoring systems may help in this regard to prevent hypoglycemia and limit excursions into hyperglycemia by providing greater information to quantify insulin sensitivity and respond faster to changes in patient condition.

Clinically, babies were observed to occasionally have periods of rapid change in sensitivity to insulin. Both increases and decreases in the level of insulin sensitivity were seen and often could not be linked to significant changes in any other routinely measured clinical variable during this time. Thus, it is possible that there were changes in some aspect of glucose metabolism that is not typically measured during neonatal intensive care. This example demonstrates robustness of the control system to account for clinically un-measurable and un-modeled effects.

The targets for glucose control vary widely between clinical units [8]. A blood glucose concentration less than 2.7 mmol/L may increase the risk of long term neurological deficiencies, and is often cited as a limit for hypoglycemia [38]. However, the precise upper limit for clinically desirable blood glucose concentration is still under debate [2,8,39]. In particular, the 8.0 mmol/L median for retrospective control may have satisfied the attending clinicians at the time and prevented attempts to lower the blood glucose further. The target range of 4–7 mmol/L selected for this study was a relatively conservative choice, reflecting the nature of these pilot trials as the first model-based study performed in premature neonates. The blood glucose target for model-based control can be readily adjusted and could thus provide a method to target specific ranges of blood glucose concentrations, without increasing the risk of hypoglycemia. This methodology could be utilized in a future randomized controlled trial to assess the efficacy of insulin infusions for glucose control whilst avoiding the complication of increased rates of hypoglycemia in the tightly controlled group.

The risk of hypoglycemia is often cited as a barrier to large-scale adoption of glycemic control by insulin infusions, especially as most neonatal hypoglycemia appears to be asymptomatic [40]. Some studies [10,41] found the incidence of hypoglycemia was significantly higher in infants receiving insulin therapy than in controls. In contrast, the results presented in this study and the adult SPRINT system developed from model-based control show a frequency of hypoglycemia similar to that seen with retrospective hospital control protocols [28]. Thus, the potential for model-based control to reduce BG levels without increasing hypoglycemia by accounting for patient variability may add another element to the discussion of ideal BG targets.

This study compared pilot trial results to retrospective data. Changes in clinical management of these infants over time may have influenced the degree of metabolic variability observed during the trials versus historical data and thus influence relative improvement in glucose control with this system. However, the low incidence of hypoglycemia is an absolute metric independent of any comparison cohort. This result suggests there is a possibility to use insulin for glycemic control without creating significant risk of hypoglycemia, provided dosing is adapted to individual, time-varying patient condition.

The stochastic model employed in this study is built from a whole-cohort perspective using data from the 21 patient retrospective group [24]. Thus, the forecasts achieve the desired prediction spread over the whole-cohort. However, the degree of variability in insulin sensitivity is patient-specific and may be linked to other clinical and diagnostic variables. Further clinical data and studies may identify patients at different stages of development or with different clinical issues. Individualized stochastic models may provide tighter forecast bands by identifying the levels of glycemic stability for individual patient.

The long-term study included two infants with gestational age of 23 weeks at birth. These infants were significantly younger than the remainder of the study populations and displayed significant resistance to insulin and persistent hyperglycemia despite insulin infusions, resulting in a clinical decision to reduce the dextrose concentration of their parenteral nutrition infusions. This result suggests that in some infants the use of insulin alone may not be enough to fully bring glycemia into control without significantly increasing the hypoglycemiarisk, and that adjusting other infusions affecting the glucose-insulin system may be necessary in these cases.

The goal of this study was to assess the efficacy of model-based insulin dosing for the control of glycemia, as opposed to eliciting the direct anabolic effects of insulin. The model-based approach can naturally modulate dextrose and insulin intake in tandem to meet nutrition goals, while controlling glycemia to allow more prospective neonatal metabolic management. This approach has already been demonstrated in adult critical care studies [27-29]. Finally, a significant range of dextrose infusions were used in these infants, and thus accounting for the total glucose load is vital to accurately choose appropriate insulin infusion rates across multiple patients.

## Conclusions

This study presents the first data using an adaptive, model-based predictive controller for insulin infusion, designed to incorporate the unique metabolic state of the neonate. The controller was used to achieve glycemic control in30premature infants weighing < 1,500 g and reduce hyperglycemia compared to retrospective hospital control without increasing hypoglycemia risk. Significant inter-patient variation in insulin sensitivity was observed, and the controller adequately managed this to regulate blood glucose concentrations. This study tested the safety of a computer system for blood glucose control and may be useful for future studies to investigate the potential impact of tight glycemic control on outcomes.

## Abbreviations

BG: Blood Glucose; IQR: Inter-Quartile Range; VLBW: Very Low BirthWeight.

## Competing interests

The authors declare they have no competing interests with respect to this study.

## Author contributions

ALC, JGC, CGP and JL developed the model-based control system and clinical software. ALC performed the data collection and results analysis AML was responsible for clinical implementation ion Christchurch Women’s Neonatal Department. GMS provided additional implementation support at Christchurch Hospital. All authors read and approved the final manuscript.

## Pre-publication history

The pre-publication history for this paper can be accessed here:

http://www.biomedcentral.com/1471-2431/12/117/prepub
